# Conference Report: INTERNATIONAL CONFERENCE ON THE CHEMISTRY OF ELECTRONIC CERAMIC MATERIALS Jackson, WY August 17–22, 1990

**DOI:** 10.6028/jres.096.003

**Published:** 1991

**Authors:** Robert S. Roth

**Affiliations:** Ceramics Division, National Institute of Standards and Technology, Gaithersburg, MD 20899

The National Institute of Standards and Technology (NIST), Ceramics Division, sponsored an international conference on the Chemistry of Electronic Ceramic Materials, supported in part by the Office of Naval Research (ONR) and the National Aeronautics and Space Administration (NASA). This was a “one of a kind” conference, deliberately held in an out-of-the-way location to stimulate informal discussion.

The conference co-chairmen were P. K. Davies, University of Pennsylvania, R. S. Roth, National Institute of Standards and Technology, and R. J. Cava, AT&T Bell Laboratories. In addition, the program committee included R. Newnham, Pennsylvania State University, M. O’Keeffe, Arizona State University, D. Smyth, Lehigh University, A. Jacobson, Exxon, and D. Kolar, Ljubljana, Yugoslavia.

The International Conference on the Chemistry of Electronic Ceramic Materials was held at the Sojourner Inn, in the Teton Village, Jackson Hole, Wyoming from August 17–22, 1990. In an attempt to maximize the development of this rapidly moving, multidisciplinary field, this conference brought together major national and international researchers to bridge the gap between those primarily interested in the pure chemistry of inorganic solids and those interested in the physical and electronic properties of ceramics. With the many major discoveries that have occurred over the last decade, one of the goals of this meeting was to evaluate our current understanding of the chemistry of electronic ceramic materials, and to assess the state of a field that has become one of the most important areas of advanced materials research.

The conference consisted of 18 invited lectures, 10 contributed verbal presentations, and 37 poster presentations. The meeting was attended by about 80 scientists from a broad spectrum of fields based at universities, industry, and national laboratories. Approximately one-third of the attendees were from outside the United States and represented 10 different countries, including Australia, Canada, China, England, France, Israel, Japan, The Netherlands, Scotland, and Yugoslavia.

The scientific presentations covered many topics including new methods for the synthesis of ceramics, the structures and properties of dielectric and ferroelectric materials, crystal chemistry, surface chemistry, high-*T*_c_ superconductors, and theory and modelling. The papers, presented either verbally or as posters, provided an up-to-date review of many aspects of these areas. For most of the verbal presentations, the discussions following the paper were successfully transcribed by a local court reporting service. These discussions served to clarify and expand certain aspects of the material covered in the lecture. Edited versions will appear in the proceedings after each paper, to be published as NIST Special Publication 804 that should appear about February 1991.

The discussion sessions were very fruitful and included repeated questions on such topics as the Fe^+3^ and Fe^+4^ ratio in various perovskites (talks by Takano, Kyoto University and Battle, Oxford University); how does one calculate dielectric constants?; what are the real radii of certain ions, and how do they substitute for each other? (talks by Shannon, DuPont; Brown, McMaster University; and O’Keeffe, Arizona State University). The major topic of discussion on high-*T*_c_ superconductors concerned the presence or absence of extra oxygen in the lattice of the Bi^+3^ containing phases (talks by Sleight, Oregon State University and Zandbergen, Delft University). Many other discussions concerned details of synthetic routes, interpretation of diffraction data, and hypothesis for future studies.

One of the most interesting and important papers presented (T. Negas, Trans. Tech.) discussed the problems of very minor impurities (<0.01%) on the microwave dielectric properties of titanate ceramics. This talk also addressed problems associated with processing parameters like hydrostatic pressing and anisotropic dielectric constants. At least five talks (Ritter, NIST; Piffard, Nantes Fr.; Payne, University of Illinois; Jacobson, Exxon; and Davies, University of Pennsylvania) were mainly concerned with low temperature synthetic routes. This topic ran throughout the conference, illustrating that these rather new techniques represent the “wave of the future” in advanced electronic ceramics, if only the costs of industrial production via these routes can be made competitive.

The success of a meeting depends on many factors ranging from the science to the weather. One of the most important is sponsorship and support. For this the conference was indebted to NIST, NASA, ONR, DuPont, AT&T Bell Laboratories, the Idaho National Engineering Laboratory (DOE), and the University of Pennsylvania. The smooth running of a meeting also depends upon considerable work by those who often receive little recognition. In this respect we were truly fortunate to have Mary Clevinger (NIST) as our conference coordinator who will also ensure that the proceedings appear in an accurate form in a timely manner. Audrey Roth and Kathy Davies organized an outstanding activities program for the many family members who attended the meeting. Denice Gilbert, Nancy Dickinson, and Tony Kostick in the Materials Science Department at the University of Pennsylvania assisted in many typing duties and several tedious budgetary matters.

It is hoped that the proceedings will contribute to the development of the Chemistry of Electronic Ceramics and, judging from the enthusiastic response of all who attended the meeting, we might all look forward to the Second International Meeting sometime in the not too distant future.

A list of the papers presented at the meeting follows:

1. Batllo, F., Cation Substitution in Barium Titanate for Dielectric Ceramic Applications.
2. Battle, P. D., Structural and Electronic Properties of Some Perovskites.
3. Billinge, S., Out of Plane Displacement of Oxygen From the CuO_2_ Sheets in Ca_0.85_Sr_0.15_CuO_2_ by Atom-Pair Distribution Function Analysis.
4. Birnie, D. P., Impurity Incorporation Mechanisms in LiNbO_3_.
5. Brese, N., Alkaline Earth Nitrides and Hydrides.
6. Bringley, J., Crystal Chemistry and Oxygen Activity Effects on T, T’ and T’ Phase Stabilities (La,RE)_2_CuO_4_ Systems.
7. Brown, I. D., Internal Strain in Perovskite Related Materials.
8. Bursill, L. A., Ferroelectric and Ferroelastic Domain Structures in Piezoelectric Ceramics.
9. Cahen, D., Room Temperature Mobility and Diffusion Coefficient of Oxygen in Polycrystalline YBa_2_Cu_3_O_7−_*_x_*.
10. Cormack, A. N., Computer Simulation Studies of Electronic Ceramics.
11. Dabrowski, B., Phase Separation in Nd_2−_*_x_*Ce*_x_*CuO_4_.
12. Davies, P. K., New Rare-Earth Cuprates with the NaCuO2 Structure.
13. Dickens, P. G., Properties of Some Mixed Uranium Oxides.
14. Feist, T. P., Soft Chemical Synthesis of Metastable Titanium, Vanadium, and Molybdenum Oxides.
15. Fitzgerald, J., Preliminary Solid-State Multi-Nuclear NMR of Titanium and Zirconium Oxide Ceramics and Precursors.
16. Fujitsu, S., Surface Energy Barrier Formed by Adsorbed Oxygen in Porous ZnO.
17. Garzon, F. H., Thermodynamic Measurements in the Y-Ba-Cu-O System.
18. Greenblatt, M., Investigations on the Structural, Electrical and Magnetic Properties of Nd_2−_*_x_*M*_x_*NiO_4+δ_,M = Ca^2+^ and Ba^2+^.
19. Huebner, W., Liquid Phase Sintering of LiF-FIuxed BaTiO_3_.
20. Ichinose, A., Synthesis and Properties of Ba-Free Superconductive (Eu,Ce)_4_(Eu,Sr)_4_Cu_6−_*_x_*M*_x_*O*_z_* (M:Fe,Co, Al).
21. Ikuma, Y., Oxygen Diffusion in Y_2_O_3_-Containing Tetragonal Zirconia Polyctystals (Y-TZP).
22. Islam, M. S., Computer Simulation of Dopant Substitution in YBa_2_Cu_3_O_7_.
23. Jacobson, A. J., Mixed Metal Oxides with the Pyrochlore Structure as Catalysis for Methane Oxidative Coupling.
24. Kao, H. C, A Correlation Between the Oxygen Stoichiometry and *T*_c_ of BiCaSrCuO Superconductors.
25. Kauzlarich, S., Synthesis and Characterization of La_1−_*_x_*Sr*_x_*TiO_3_ (0⩽*x*⩽0.05).
26. Kolar, D., Chemical Reaction Controlled Microstructures and Properties of Ferroelectric Ceramics.
27. Lane, C, Molecular Dynamics Simulations of Ion Motion in Divalent and Mixed Monovalent-Divalent Beta″-Alumina.
28. Lightfoot, P., Excess Oxygen Defects in Layered Cuprates.
29. Marezio, M., Oxygen Stoichiometiy and Superconducting Properties in High-*T*_c_ Cu-Based Oxide Superconductors.
30. Nasrallah, M., Polymeric Synthesis of Perovskite Powders and Films.
31. Nath, A., Can Co(Fe) Substituent in YBa_2_Cu_3_O_7−δ_ Migrate Back and Fortli Between Cu(l) and Cu(2) Sites?
32. Navrotsky, A., Calorimetric Studies of Ceramics.
33. Negas, T., Chemistry and Properties of Temperature Compensated Microwave Dielectrics.
34. Newnham, R. E., Tunable Transducers: Nonlinear Phenomena in Electroceramics.
35. Norton, M., Electrocrystallization in the Ba-K-Bi-O System.
36. Nowotny, J., Surface Properties of BaTiO_3_ at Elevated Temperatures.
37. O’Keefe, M., Empirical Methods in Crystal Chemistry.
38. Payne, D. A., Synthesis of Electrical Ceramics by Polymeric Alkoxide Routes.
39. Piffard, Y., Ion Exchange Reactions of Layered Phosphatoantimonic Acids: A Route for New Catalysts and Luminescent Materials.
40. Poeppelmeier, K., Structural Diversity in Oxygen-Deficient Perovskites.
41. Rawn, C, Phase Equilibria and Crystal Chemistry in the SrO-CaO-Bi_2_O_3_-CuO Systems.
42. Ritter, J. J., Molecular Chemistry and the Synthesis of Precusors to Electronic Ceramic Materials.
43. Rohrer, G. S., A Scanning Tunneling Microscopy Study of Single Crystal ZnO and TiO_2_ Surfaces.
44. Rosenfeld, D., A Real Space Analysis of Short Range Order in Ferroelectric Pb(Mg_1/3_Nb_2/3_)O_3_.
45. Roth, R. S., Synthesis and Characterization of Phases in the System Ba-Au-Ag-O.
46. Roth, S., Pollution Control Catalysts: Materials Design Considerations.
47. Sasaki, Y., Mechanism of PNN Based Perovskite Ceramics Formation.
48. Scott, B. A., New LaCuO_3−δ_ Perovskites Prepared at High Oxygen Pressure.
49. Shannon, R. D., Factors Determining the Dielectric Constants of Oxides and Fluorides.
50. Shrout, T. R., Conventionally Prepared Submicron ElectroCeramic Powders by Reactive Calcination.
51. Sleight, A. W., Crystal Chemistry of Oxide Superconductors.
52. Smyth, D. E., A Structural Basis for the Different Directions of Oxygen Nonstoichiometry in La_2_CuO_4_ and Nd_2_CuO_4_.
53. Steinfink, H., Ruddlesden-Popper Phases A*_n_*_+1_MnO_3n+1_ Structures and Properties.
54. Sunshine, S., Structure and Properties of Reduced Early Transition Metal Oxide Single Crystals Grown from Borate Fluxes.
55. Sunstrom, J., Magnetic and Electronic Properties of A*_x_*Ce_1−_*_x_*TiO_3+y_(A = Sr, Ba) (0<*x* <.5).
56. Switzer, J. A., Electrodeposition of Nanomodulated Electronic Ceramic Thin Films.
57. Takano, M., Solid State Chemistry of Perovskite and Related Oxides of Fe^4+^, Ni^3+^, and Cu^2+^.
58. Thompson, M., Kinetics and Mechanism of the Formation of Mg_2_Al_4_Si_5_O_18_ from MgAl_2_O_4_ and SiO_2_ in a Bismuth Oxide Flux.
59. Trontelj, M., Influence of Sb_2_O_3_ on the Sintering of ZnO Ceramics.
60. Wuensch, B. J., Structure-Property Relations in Close-Packed Fast-Ion Conductors.
61. Zandbergen, H., Microstructures in High Temperature Superconductors.

